# Holo-imprinting polarization optics with a reflective liquid crystal hologram template

**DOI:** 10.1038/s41377-022-00746-3

**Published:** 2022-03-10

**Authors:** Jianghao Xiong, Qian Yang, Yannanqi Li, Shin-Tson Wu

**Affiliations:** College of Optics and Photonics, University of Central Florida, Orlando, FL 32816 USA

**Keywords:** Imaging and sensing, Displays

## Abstract

Liquid crystal polarization optics based on photoalignment technique has found pervasive applications in next-generation display platforms like virtual reality and augmented reality. Its large-scale fabrication, however, remains a big challenge due to the high demands in small feature size, fast processing speed, and defects-free alignment quality during the photoalignment process, especially for large-angle reflective devices. Here we propose a new concept of holo-imprinting based on non-contact replication of polarization pattern with a reflective liquid crystal hologram as a template. Our theoretical analysis and experimental results validate the possibility of generating a high-quality polarization pattern exploiting the self-interfering beams of reflective holograms. The method can be extended to numerous devices, from transmissive to reflective, from small angle to large angle, and from grating, lens, to freeform optics. Its widespread impact on the fabrication of liquid crystal polarization optics for advanced display and imaging systems is foreseeable.

## Abstract (web summary)

Holo-imprinting of photo-aligning planar liquid crystal optics enables a fast and efficient fabrication process for advanced imaging and display devices, including augmented reality and virtual reality.

## Graphical abstract

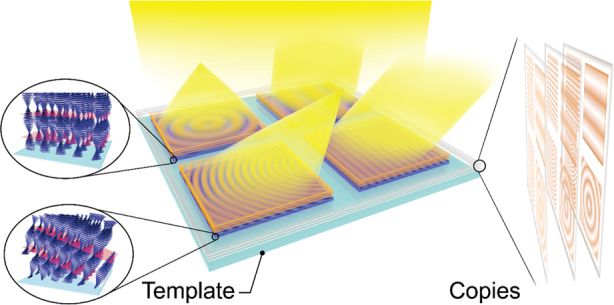

## Introduction

Polarization optics based on photo-aligned liquid crystal (LC) enables the versatile modulations of light with various functionalities and dynamic tunability, from transmissive/reflective diffractive optical elements^1–3^, to self-organized soft photonic crystals^4,5^ and stimuli-response devices^4,6^. Unlike traditional holographic optical elements based on bulk-recording the interfering electric fields, such a LC polarization optics records the electric field information in a nanometer-thick photoalignment layer. The LC layer (only a few microns) placed atop self-assembles into a functional optical layer with excellent image quality and diffraction efficiency. Such an ultra-thin LC film is extremely attractive for optical systems pursuing a compact form factor. A great example is near-eye displays like virtual reality (VR) and augmented reality (AR), where the headset should be lightweight and compact while delivering high-performance images^7^. The LC alignment methods have evolved from early mechanical buffing to non-contact photoalignment based on linear photo-polymerization^8,9^ for high-resolution multi-domain liquid crystal displays (LCDs), then to azo-dye-based photoalignment for polarization holography^2,10^. Due to the simple fabrication process and excellent alignment quality, azo-dye-based photoalignment has lately been extensively adopted to fabricate various types of LC polarization optics^2^.

Transmissive LC polarization optics based on geometric phase, or Pancharatnam–Berry phase, has a relatively long history, almost accompanying the early development of azo-dye-based photoalignment methods^11,12^. Due to the limited LC self-assembly capability^13,14^, such a transmissive LC device usually entails a relatively small diffraction angle, which can still be adopted in VR to achieve resolution enhancement^15^ or depth modulation^16^. On the other hand, reflective LC polarization optics^17–22^ has only been explored recently. For devices with a large alignment period, cholesteric liquid crystal (CLC) can still follow the bottom alignment in a planar form, establishing the so-called Bragg-Berry devices^17,20,23^. However, when the alignment period becomes small and comparable to the CLC pitch, the tilted helical structure will be formed^22^. The totally different self-assembly mechanism from its transmissive counterpart leads to a very large diffraction angle and even entrapment of light into total internal reflections. Therefore, it has since found wide applications in VR and AR, such as holographic lens in pancake optics for VR^24^, couplers in waveguide displays^25^, and dynamic pupil-steerable combiners in Maxwellian displays^26^ for AR.

Despite the above-mentioned attractive properties, the actual adoption of these photo-aligned LC polarization optics for widespread applications requires a practical fabrication method for large-scale production. The key fabrication bottlenecks are photo exposure and LC deposition. The latter is compatible with matured roll-to-roll film production flow^27^ (for LC polymeric film) and large-scale LC infiltration^28^ (for cell-type components) in the traditional LCD production line. Therefore, the major challenge lies in the exposure step. Numerous types of exposure methods have been proposed. One commonly adopted method is based on holographic exposure with an interferometer^29^, where a coherent light beam is split into two arms and then recombined to form an interference pattern. While it is suitable for the laboratory-scale fabrication of small-area samples, further scaling up the exposure system may be costly and difficult. Another approach is to write the polarization pattern with a scanning laser^30^. This method has a large degree of freedom, but the slow writing speed prevents the possibility of mass production. Besides, the minimum pattern feature is about the size of a focused laser spot, which is about hundreds of nanometers. Such a feature size may be adequate for transmissive photo-aligned LC polarization optics, but is still too large for a reflective device, whose pattern size is usually in the order of ~10 nm to achieve a large diffraction angle. So far, imprinting methods based on a master template possess the highest potential for mass production. Traditional nano-imprinting technique based on patterned groove has been demonstrated^31^. Nonetheless, aside from lower alignment quality, to fabricate a template with sub-wavelength groove feature size is also challenging. Optical masks based on transmissive polarization optics^32^, plasmonic photopatterning^33,34^, or absorptive dye^35,36^ are also proposed. While the fabrications of large-period (micrometer-scale) transmissive LC polarization optics have been demonstrated, their feasibility in producing reflective devices with sub-wavelength feature size remains unclear.

**Fig. 1:**
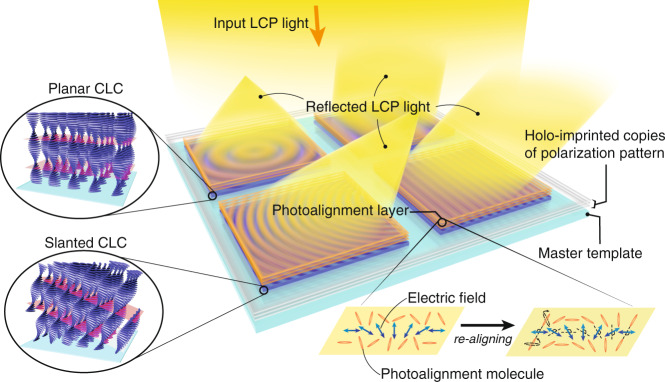
Concept of the holo-imprinting method. The polarization patterns of an on-axis lens, a small-angle grating, an off-axis lens, and a large-angle grating on the master template are reproduced by the input LCP light and reflected LCP light. Substrates with a photoalignment layer are placed in the reproduced polarization pattern to produce holo-imprinted copies

Here a new approach for fabricating photo-aligned LC polarization optics based on optical imprinting is proposed. As sketched in Fig. 1, a master template consists of high-efficiency reflective CLC polarization holograms. Depending on the pattern period, the CLC directors may exhibit a planar structure (large period) or tilted structure (small period). The input circularly polarized (CP) light, e.g., a left-handed circularly polarized (LCP), with the same handedness as the CLC will be reflected with nearly 100% diffraction efficiency if the wavelength and incident angle are within the reflection bands. The reflected light and input light together form the electric field pattern identical to the one recording the template. The pattern can be used to replicate the photoalignment layers with excellent optical quality and exposure speed. We refer to this method as *holo-imprinting* due to its self-interfering feature and high processing speed like nanoimprinting.

## Results

The concept of copying holograms has been proposed for intensity-based holograms a long time ago^37^. The spatially variant field intensity can be reproduced by controlling the diffraction efficiency of the master hologram. However, the situation is more complicated for the photoalignment process because of the special property of photoalignment molecules. The polarization pattern instead of field intensity requires more attention here. The rod-shaped azo-dye molecules for photoalignment tend to align perpendicular to the long axis of a general elliptically polarized light, where the absorption is minimum. Therefore, a patterned linearly polarized light has the best alignment quality. To generate the sinusoidal linear polarization pattern, traditional interferometer methods use two CP lights with opposite handedness incident on the same side of the sample. However, it is also possible to generate the linear polarization pattern with two CP lights with the same handedness by changing the incident configurations.

**Fig. 2:**
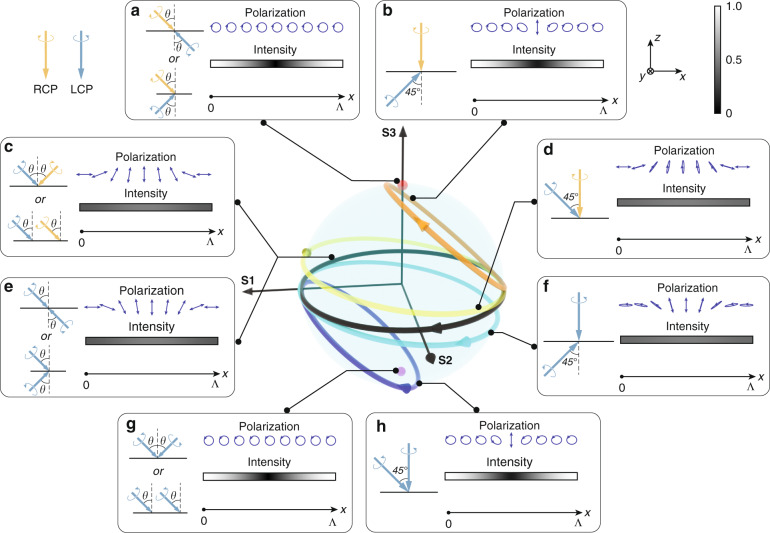
Interference of two CP lights. Polarization patterns of two interfering CP lights are plotted on Poincaré sphere. The curves correspond to different configurations and polarization states. Each subfigure corresponds to a curve on the sphere with one or more possible configurations. The left side of each subfigure is the light incidence configuration. The right side is the polarization and intensity pattern within one pattern period Λ. The scale bar in the upper right corner indicates the light intensity in each subfigure. **a** LCP and RCP lights and **e** two LCP lights with opposite incidence and incidence symmetric to the surface. RCP light at normal incidence and LCP light at 45° incidence on **b** opposite side and **d** same side. **c** LCP and RCP lights and **g** two LCP lights with same incidence and incidence symmetric to the surface normal. Two LCP lights with one normal incidence and the other 45° incidence on **f** opposite side and **h** same side

For better clarity, the interfering polarization pattern of two CP beams with different polarization states and incident configurations are plotted in Fig. 2. The calculation is based on adding up the electric field in *x*–*y* plane and converting it to Stokes parameters. The spatially periodic polarization pattern for each configuration forms a closed curve on Poincaré sphere. The equator corresponds to a perfect linear polarization state with the best alignment quality. Curves closer to the equator (with a smaller S3 component) have a better alignment quality. Each curve in Fig. 2 corresponds to one or several configurations illustrated in the box, where the left side represents light incidence configurations, and the right side is the polarization pattern and intensity distribution within one pattern period Λ. Note that it is unlikely to plot all the configurations in one graph. Therefore, only some representative ones are chosen here. For opposite-handed CP lights incident from opposite sides, all configurations produce a polarization state with a large S3 component and nonuniform light intensity, which amounts to poor alignment quality. Figure 2a depicts the cases of symmetric (to surface normal) incidence and opposite incidence. The curve degenerates to a point in the north pole, with a sinusoidal intensity pattern. For RCP light at normal incidence and LCP light incident from the opposite side at 45° angle (Fig. 2b), the curve still has a large S3 component. The polarization and intensity patterns further reveal that most of the pattern has a large S3 component, except for a small region in the center with weak light intensity. On the other hand, for CP lights with opposite handedness and same-side incidence, all configurations produce an excellent alignment quality. For example, the cases of symmetric incidence and identical incidence from the same side (Fig. 2c) produce a perfect linear polarization pattern, with uniform light intensity. For RCP light at normal incidence and LCP light at 45° incidence (Fig. 2d), the pattern still exhibits good quality, with a small S3 component. For the same-handed CP lights, the situation is reversed. All configurations with opposite-side incidence generate an excellent polarization pattern for alignment and vice versa. Detailed calculation and discussion can be found in Supplementary S1.

**Fig. 3:**
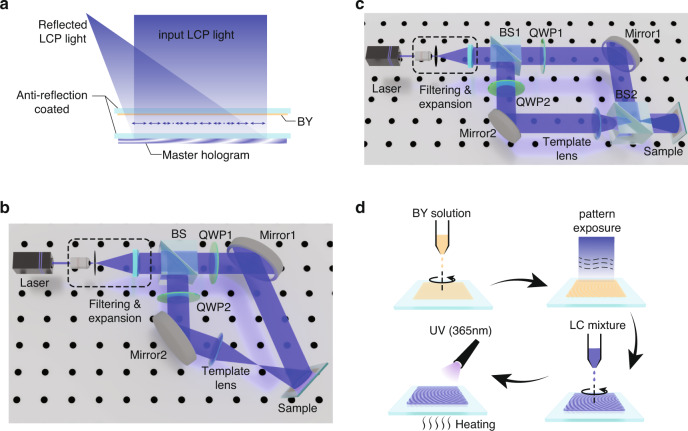
Fabrication process. **a** Configuration of holo-imprinting process. Sketch of interferometers for the master template fabrication for **b** large-angle devices and **c** small-angle devices. The laser after spatial filtering and expansion is split into two arms by a beam splitter (BS), passing through a quarter-wave plate (QWP) in each arm to be converted to LCP and RCP lights, and finally recombined. **d** Detailed fabrication flow of the whole process, including the spin-coating of BY, pattern exposure, spin-coating of LC mixture, and UV polymerization

To generate two CP lights with the same handedness and opposite incident directions, a standing-wave interferometer using a mirror-lens reflector has been demonstrated^38^. But the fabricated sample still has a relatively small diffraction angle (around 5°) and the feasibility to scale up the whole fabrication remains unclear. Alternatively, a reflective photo-aligned LC hologram can also serve this purpose, with excellent thinness for large-scale production and wide-range diffraction angle. As depicted in Fig. 3a, the master hologram reflects the incident LCP light and forms the original polarization field on the incident side. When a sample coated with a photoalignment material (Brilliant Yellow (BY)) is placed above the master hologram, the pattern can be copied onto the sample. Glass substrates with anti-reflection coating on one side are used here to reduce Fresnel reflections that would otherwise degrade the quality of reproduced polarization field. In the experiment, placing the BY layer upwards or downwards produces similar results. To fabricate a master hologram, two types of interferometers for pattern exposure, as depicted in Fig. 3b, c, are adopted to fabricate the large-angle and small-angle samples, respectively. In our experiment, four types of templates are fabricated, namely a large-angle reflective grating, an off-axis reflective lens, a small-angle reflective grating, and an on-axis lens. The first two adopt the interferometer in Fig. 3b and the last two in Fig. 3c. The difference in the interferometer set-up between grating and lens fabrications involves whether to insert the template lens and adjustment of mirror’s rotation angle.

The whole procedure is plotted in Fig. 3d. The BY solution is firstly spin-coated on a pre-cleaned glass substrate. The sample then undergoes the exposure process (Fig. 3b, c) which forms a patterned BY layer. The LC mixture is later spin-coated on the sample, forming the functional LC polarization optics. Instead of traditional fluidic LCs, a reactive mesogen (RM) is used in this work for the fabrication of both templates and samples. RMs have similar properties to LCs; they can form a liquid crystalline phase after the evaporation of solvent material^39^. The end reactive groups crosslink under ultraviolet (UV) polymerization process, which in turn stabilize the liquid crystalline phase. Here the fabrication procedures of the master hologram and copied hologram are similar, except for the difference in the pattern exposure process and composition of the LC mixture. The samples are finally placed on a heating stage and undergo UV illumination for polymerization. The laser employed has a wavelength of 457 nm. For master holograms, the efficiency should be high at the laser wavelength (457 nm) for the later holo-imprinting process. Therefore, the film thickness and reflection band are finely adjusted to produce >95% efficiency at 457 nm.

**Fig. 4:**
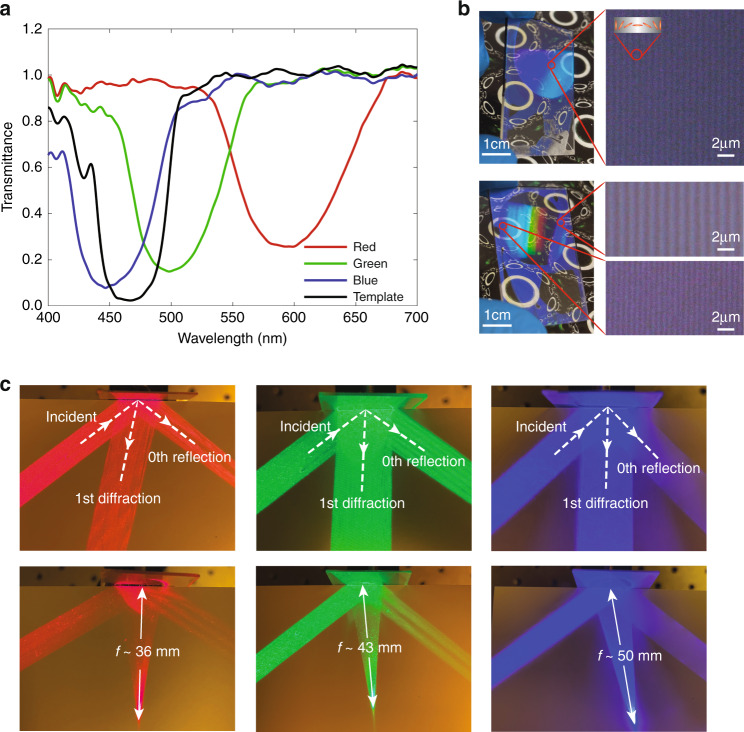
Large-angle devices. **a** Transmission curves of the large angle grating template and RGB samples. **b** Photos and POM images (×100) of templates of the large-angle grating (upper) and off-axis lens (lower). **c** Photos of RGB samples diffracting light. The grating and lens patterns are the same for RGB samples, resulting in different diffraction angles for grating and focal lengths for the lens

For a large-angle reflective grating and off-axis reflective lens, once the polarization pattern is replicated from the template to the sample, the deposition of the LC mixture can have a certain degree of freedom. In our experiment, the concentration of chiral dopant in the LC mixture is adjusted so that three sets of devices with diffraction bands centered at red (R), green (G), and blue (B) wavelengths are fabricated separately. The angle between recording beams for both grating and off-axis lens is 45°. The focal length of the template lens for off-axis lens devices is 50 mm. The transmission bands of four grating devices (RGB and template) are shown in Fig. 4a. The template grating exhibits an efficiency of about 97% at 457 nm, with a measured thickness of about 2.3 µm using a profilometer. The imprinted RGB samples have a thickness of around 2.0 µm and central wavelengths around 450, 500, and 600 nm, respectively. For the off-axis lenses, the transmission bands are similar, except that a small band shift occurs when measuring different sample locations. This is caused by the slightly different Bragg angle due to the change of alignment period.

Photos and polarized optical microscopic (POM) images of templates are shown in Fig. 4b. The background is a printed image of a series of small ellipses surrounding large circles, under the office lighting condition of two rectangular lamps and illuminance of 640 lux. The good visibility of the background image through the template sample implies great see-through ability. For the grating, the image of the rectangular ceiling lamp above the camera is reflected and deflected and appears in the sample region as a bright blue region. For the lens, the lamp image is reflected, deflected, and minified, accompanied by color dispersion due to the diffractive nature of the sample. The fringe period is equal to the alignment optical pitch where the azo-dye molecules rotate 180°. The grating has a period of around 0.65 µm, which agrees with the calculated value of 0.6 µm. For the off-axis lens, the period varies from around 0.49 µm to around 1.2 µm, due to the spatially variant diffraction angle. For a rough estimation of feature size, a 10° division for one period of the grating (180°, 0.6 µm) leads to a feature size of around 30 nm. The small feature size further confirms our previous claim of the fabrication difficulty of large-angle devices by other approaches. The pictures of RGB samples diffracting light are shown in Fig. 4c. For the grating, because the alignment pattern is fixed, the incident light with a longer wavelength (RG) than the recording one (B) shows a larger diffraction angle. For the off-axis lens, the change in wavelength also leads to a focal length shift. The focal length varies from the recording focal length (50 mm) for the blue light, to 43 mm for the green light, and 36 mm for the red light, which basically agrees with the inherent dispersion relation.

**Fig. 5:**
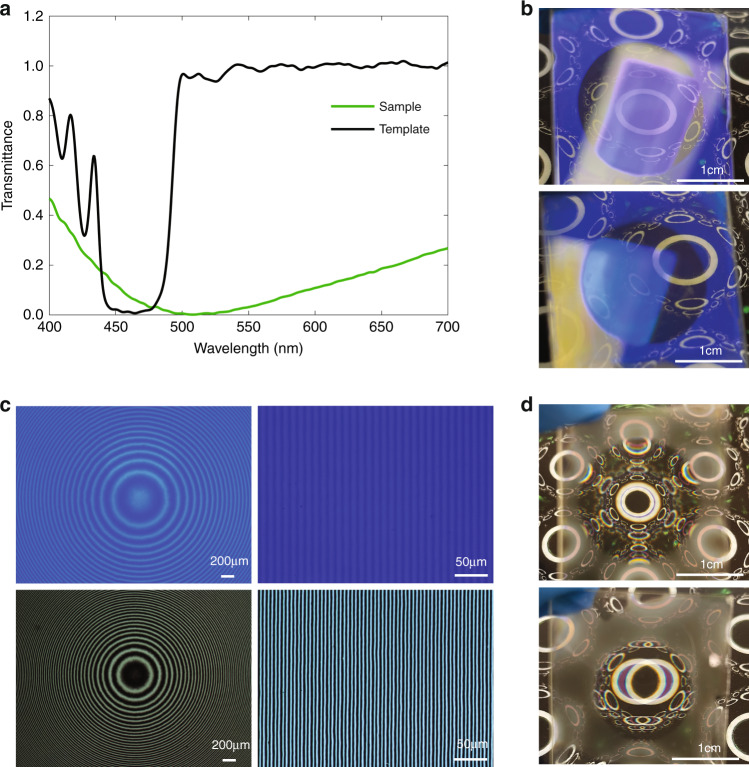
Small-angle devices. **a** Transmission curves of the small-angle grating template and transmissive sample. **b** Photos of grating and lens templates. The minified and slighted deflected images of the ceiling lamp can be observed for the lens and grating. **c** POM images (×5 for left and ×20 for right) of templates (top) and transmissive samples (bottom). **d** Photos of transmissive samples diffracting background light for the lens (upper) and grating (lower). The magnified and minified images correspond to ±first diffraction orders for the lens. The left and right deflected images correspond to ±first diffraction orders for the grating

For the on-axis templates (small-angle grating and on-axis lens), the imprinted samples are used to fabricate transmissive devices, because there is no worry of LC self-assembly for a larger alignment period. It should be noted, however, the fabrication of reflective devices is also possible simply by using a LC mixture with a high chiral concentration. The film thickness is around 2.3 µm for the template and 1.5 µm for the sample. The template lens in the interferometer has a focal length of 200 mm. The transmission curves of the grating sample and template are plotted in Fig. 5a. The template exhibits an efficiency of around 99% at 457 nm. The transmission curve of the sample indicates the zeroth-order light leakage, which can be regarded as diffraction efficiency subtracted from unity. The efficiency is the highest (~100%) for green light but decreased to around 85% for red and blue. It is worth pointing out that a broadband transmissive device can be achieved by employing a multi-twist structure^40–42^, which is compatible with the patterning technique demonstrated here. Photos of the templates are shown in Fig. 5b. The rectangular ceiling lamp forms a minified image for the lens and a slightly deflected image for the grating. The POM images of templates and samples are shown in Fig. 5c. Notice the fringe period of the template is twice that of the sample. This is because the dark-bright fringe period in the sample corresponds to when a half-wave plate rotates 90°. The photos of the transmissive devices diffracting see-through light are shown in Fig. 5d. The unpolarized light from the background image is diffracted into the ±first-order LCP/RCP light. For the lens, the ±first orders form magnified and minified images, which correspond to larger and smaller circles in the center. For the grating, the orders from the left image and right image overlap with each other. Notice the chromatic dispersion of the circles is easily observed, which originates from the diffractive nature of the samples.

## Discussion

For the feasibility study, the size of our fabricated master holograms is only ~1 cm in diameter, a much larger master hologram for mass-production can be readily obtained by combining the interferometer with a translation stage. In most applications, the pattern size of an individual optical element may be in the order of several centimeters, like, for example, the out-coupler grating for a waveguide AR display combiner, or the combiner lens for Maxwellian-AR display. Therefore, a master hologram with an array of identical optical patterns can be easily fabricated by the repeated move-and-expose steps. For proof-of-concept, we fabricated a 2-inch template with a 2-by-2 array of grating and lens patterns and used it for holo-imprinting a 2-inch sample. Details are described in Supplementary S2. For some applications demanding a large continuous pattern, a high-precision translation stage and an arbitrary pattern generator like a spatial light modulator^43^, freeform surface^44^ or laser scanning^30^ may be required. Additionally, although only *passive* devices based on LC polymer film are fabricated in this paper, our holo-imprinting technique is also compatible with the fabrication of *active* (switchable) LC cells, by replacing the LC polymer spin-coating step with LC infiltration. Because one side of the alignment layer can produce an optical component with good quality as demonstrated, the LC cell formation can be performed after the pattern exposure process of one substrate, which eliminates the potential surface reflection from the LC cell.

For large-scale manufacturing, the light source also needs extra attention. Here a laser with a long coherence length (>100 m) is used for the fabrication of both templates and samples. Typically, a high-power laser usually entails a shorter coherence length. Considering the distance between the sample and template is about several millimeters, a laser with a coherence length longer than one centimeter is preferred. Additionally, the laser wavelength can also be changed by adopting a different photoalignment material with matched absorption bands.

In conclusion, we propose and demonstrate a new non-contact holo-imprinting approach for fabricating large-scale LC polarization optics, based on self-interference of reflective polarization holograms. The validity of the method is proven by analyzing the polarization pattern of CP light interference and by experiment. The method can accommodate both large-period patterns and large-angle diffraction patterns with subwavelength-scale feature sizes, which has not been demonstrated in previous approaches like nanoimprinting or absorption-mask approaches. Its widespread application in the fabrication of LC polarization optics for advanced display and imaging systems is foreseeable.

## Materials and methods

### Materials

BY (from Tokyo Chemical Industry Co., Ltd.) is dissolved in *N*,*N*-dimethylformamide with a weight concentration of 0.2%. The BY solution is filtered through a 0.2-µm Teflon syringe filter to eliminate impurities. The LC mixture consists of solvent toluene and precursor. The precursor mainly contains LC monomer RM257 and chiral dopant S5011, purchased from Jiangsu Hecheng Advanced Materials Co., Ltd. and photo-initiator Irgacure 651 from BASF. The helical twisting power of S5011 in RM257 is 131 µm^−1^. Changing the concentration of S5011 in the precursor leads to a different helical pitch and consequently diffraction band. Additionally, a surfactant Zonyl 8857A (from DuPont) is added into the precursor with a weight concentration of 0.1%. The surfactant increases the surface tension at the LC-air interface and changes the homeotropic anchoring to degenerate planar anchoring, which helps improve the LC alignment quality. The detailed material compositions for different devices can be found in Table S1.

### Fabrication

The glass substrate with one side of anti-reflection coating (<0.5% at 457 nm) is provided by Goertek Electronics. The substrate is cleaned by ethanol, acetone, and isopropyl alcohol, and then treated by UV-Ozone for 10 min. The BY solution is spin-coated onto the substrate at 500 r.p.m. for 5 s and 3000 r.p.m. for 30 s. The humidity of the environment for spin-coating BY is controlled below 30% relative humidity^45^. Then the sample experiences the exposure process. The laser (Cobolt Twist^TM^) has an output power of 200 mW. The exposure dosage is about 6 J/cm^2^ (35 mW/cm^2^ for 3 min). The importance of humidity control in the exposure process is found to be insignificant. After that, the LC mixture is spin-coated onto the sample for 30 s. The spinning speed is 1200 r.p.m. for templates, 1500 r.p.m. for reflective samples, and 2000 r.p.m. for transmissive samples. After the deposition of LC material, the sample is placed on a heating stage (80 °C) for 5 min to evaporate the remaining solvent and relax LC molecules for better alignment quality. Finally, UV light exposure is performed in nitrogen-rich environment to polymerize the sample, with a dosage of 3 J/cm^2^ (10 mW/cm^2^ for 5 min).

### Measurement

The transmittance curve is measured with Ocean Optics spectrometer model HR4000CG-UV-NIR, paired with Edmund BDS130 light source. A left-handed circular polarizer is placed in the light path before the sample. For measurement of transmittance curve for transmissive samples, normally a right-handed circular polarizer should be inserted after the sample to eliminate diffracted light. But in our case, it is omitted because the grating diffraction angle is large so that the diffracted light does not enter the detector. POM images are observed with an Olympus BX51 microscope. The microscope light illuminates the reflective devices from the upside and the transmissive ones from the downside.

## Supplementary information

Supplementary Information

## Data Availability

All data needed to evaluate the conclusions in the paper are present in the paper. Additional data related to this paper may be requested from the authors.

## References

[1] Chen P (2020). Liquid-crystal-mediated geometric phase: from transmissive to broadband reflective planar optics. Adv. Mater..

[2] Xiong JH, Wu ST (2021). Planar liquid crystal polarization optics for augmented reality and virtual reality: from fundamentals to applications. eLight.

[3] Nersisyan SR (2010). The promise of diffractive waveplates. Opt. Photonics News.

[4] Zheng ZG (2017). Light-patterned crystallographic direction of a self-organized 3D soft photonic crystal. Adv. Mater..

[5] Zheng ZG (2016). Three-dimensional control of the helical axis of a chiral nematic liquid crystal by light. Nature.

[6] Zola RS (2019). Dynamic control of light direction enabled by stimuli-responsive liquid crystal gratings. Adv. Mater..

[7] Xiong JH (2021). Augmented reality and virtual reality displays: emerging technologies and future perspectives. Light.: Sci. Appl..

[8] Schadt M (1992). Surface-induced parallel alignment of liquid crystals by linearly polymerized photopolymers. Jpn. J. Appl. Phys..

[9] Schadt M, Seiberle H, Schuster A (1996). Optical patterning of multi-domain liquid-crystal displays with wide viewing angles. Nature.

[10] Chigrinov, V. G., Kozenkov, V. M. & Kwok, H. S. *Photoalignment of Liquid Crystalline Materials: Physics and Applications* (Wiley, 2008).

[11] Chen AGS, Brady DJ (1992). Surface-stabilized holography in an azo-dye-doped liquid crystal. Opt. Lett..

[12] Gibbons WM (1991). Surface-mediated alignment of nematic liquid crystals with polarized laser light. Nature.

[13] Komanduri RK, Escuti MJ (2007). Elastic continuum analysis of the liquid crystal polarization grating. Phys. Rev. E.

[14] Sarkissian H (2006). Periodically aligned liquid crystal: potential application for projection displays. Mol. Cryst. Liq. Cryst..

[15] Lee YH, Zhan T, Wu ST (2017). Enhancing the resolution of a near-eye display with a Pancharatnam-Berry phase deflector. Opt. Lett..

[16] Zhan T, Lee YH, Wu ST (2018). High-resolution additive light field near-eye display by switchable Pancharatnam–Berry phase lenses. Opt. Express.

[17] Kobashi J, Yoshida H, Ozaki M (2016). Planar optics with patterned chiral liquid crystals. Nat. Photonics.

[18] Lee YH, He ZQ, Wu ST (2019). Optical properties of reflective liquid crystal polarization volume gratings. J. Optical Soc. Am. B.

[19] Lee YH, Yin K, Wu ST (2017). Reflective polarization volume gratings for high efficiency waveguide-coupling augmented reality displays. Opt. Express.

[20] Rafayelyan M, Brasselet E (2016). Bragg-Berry mirrors: reflective broadband q-plates. Opt. Lett..

[21] Weng YS (2016). Polarization volume grating with high efficiency and large diffraction angle. Opt. Express.

[22] Xiong JH, Chen R, Wu ST (2019). Device simulation of liquid crystal polarization gratings. Opt. Express.

[23] Rafayelyan M, Tkachenko G, Brasselet E (2016). Reflective spin-orbit geometric phase from chiral anisotropic optical media. Phys. Rev. Lett..

[24] Maimone A, Wang JR (2020). Holographic optics for thin and lightweight virtual reality. ACM Trans. Graph..

[25] Weng YS (2018). Liquid-crystal-based polarization volume grating applied for full-color waveguide displays. Opt. Lett..

[26] Xiong JH (2021). Aberration-free pupil steerable Maxwellian display for augmented reality with cholesteric liquid crystal holographic lenses. Opt. Lett..

[27] Søndergaard RR, Hösel M, Krebs FC (2013). Roll-to-Roll fabrication of large area functional organic materials. J. Polym. Sci. B Polym. Phys..

[28] Souk, J. et al. *Flat Panel Display Manufacturing* (Wiley, 2018).

[29] Crawford GP (2005). Liquid-crystal diffraction gratings using polarization holography alignment techniques. J. Appl. Phys..

[30] Kim J (2015). Fabrication of ideal geometric-phase holograms with arbitrary wavefronts. Optica.

[31] He ZQ (2018). Switchable Pancharatnam-Berry microlens array with nano-imprinted liquid crystal alignment. Opt. Lett..

[32] Nersisyan SR (2009). Characterization of optically imprinted polarization gratings. Appl. Opt..

[33] Jiang M (2019). Low f-number diffraction-limited Pancharatnam–Berry microlenses enabled by plasmonic photopatterning of liquid crystal polymers. Adv. Mater..

[34] Jiang M (2018). Liquid crystal Pancharatnam–Berry micro-optical elements for laser beam shaping. Adv. Optical Mater..

[35] Li Y (2019). Single-exposure fabrication of tunable Pancharatnam-Berry devices using a dye-doped liquid crystal. Opt. Express.

[36] Zhan T (2020). Absorption-based polarization gratings. Opt. Express.

[37] Harris FS, Sherman GC, Billings BH (1966). Copying holograms. Appl. Opt..

[38] He ZQ, Yin K, Wu ST (2020). Standing wave polarization holography for realizing liquid crystal Pancharatnum-Berry phase lenses. Opt. Express.

[39] Yun CJ, Song JK (2017). Functional films using reactive mesogens for display applications. J. Inf. Disp..

[40] Oh C, Escuti MJ (2008). Achromatic diffraction from polarization gratings with high efficiency. Opt. Lett..

[41] Zou JY (2020). Broadband wide-view Pancharatnam-Berry phase deflector. Opt. Express.

[42] Gao K (2017). High-efficiency large-angle Pancharatnam phase deflector based on dual-twist design. Opt. Express.

[43] Wu H (2012). Arbitrary photo-patterning in liquid crystal alignments using DMD based lithography system. Opt. Express.

[44] Jang C (2020). Design and fabrication of freeform holographic optical elements. ACM Trans. Graph..

[45] Wang JR (2017). Effects of humidity and surface on photoalignment of brilliant yellow. Liq. Cryst..

