# Supplementary material for: Holo-imprinting polarization optics with a reflective liquid crystal hologram template
Source: Light Sci Appl. 2022 Mar 10;11:54. doi: 10.1038/s41377-022-00746-3 (PMC8913690; doi:10.1038/s41377-022-00746-3)
Supplement: Supplementary file 1 — Supplementary Information [file 41377_2022_746_MOESM1_ESM.docx]

**Supplementary Information for Holo-imprinting polarization optics with a reflective liquid crystal hologram template**

*Jianghao Xiong, Qian Yang, Yannanqi Li and Shin-Tson Wu^*^*

*College of Optics and Photonics, University of Central Florida, Orlando, FL 32816, USA*

*Corresponding author: [swu@creol.ucf.edu](mailto:swu@creol.ucf.edu)

_____________________________________________________________________________

**S1 Calculation of interference polarization pattern**

The electric field in *x*-*y* plane for an obliquely incident CP light in Fig. S1 can be expressed as:

$\overrightarrow{E}=e^{i\overrightarrow{\mathbf{k}}\cdot\overrightarrow{\mathbf{r}}}(\widehat{\mathbf{x}}'\pm i\widehat{\mathbf{y}}')=e^{-ik_{0}x\sin\theta}(\cos\theta\widehat{\mathbf{x}}\pm i\widehat{\mathbf{y}}\pm\sin\theta\widehat{\mathbf{z}})$ (S1)


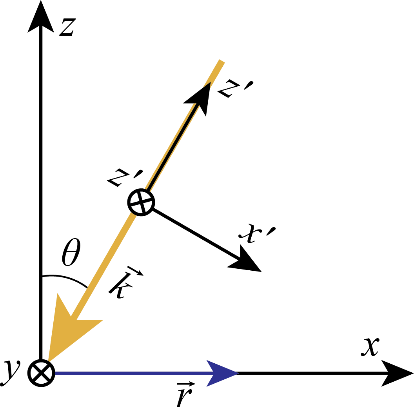


Fig. S1. Illustration of light incident configuration

For the interference of two CP lights, we simply add their electric fields together. Several points need to be noted here. The first is that the z-component of electric field (*E_z_*) is neglected in the calculation. This is mainly for the consideration of the aligning property of azo dyes. Because the azo-dye molecules tend to align perpendicular to the electric field. As long as they stay in the *x*-*y* plane, they are always perpendicular to *E_z_*. Second, the addition of two periodic fields does not necessarily lead to a periodic one. This means the curve on the Poincaré sphere is not precisely a closed loop. However, from the calculations we find that the fluctuation around the main curve is small. Therefore, we plot the curve without considering the fluctuations. Third, in some cases the pattern period reaches infinity, like, for example, when the light incident angle is the same for the interference of LCP and RCP lights. Under such a condition, the curve on Poincaré sphere should be interpreted as the limit-approaching result for infinitesimal difference of incident angle.

One may wonder why the interference of two LCP lights produces other polarization states. We shall give a detailed discussion here. Consider a simple case of two LCP lights with one normal incidence and one with incident angle *θ*. The jones vector of the interfering field can be expressed as

$J\text{\textasciitilde}\left[\begin{matrix} 1+\cos\theta e^{-ik_{0}x\sin\theta} \\ i(1+e^{-ik_{0}x\sin\theta}) \end{matrix}\right]$ (S2)

Here, the normalization factor is ignored. Within one pattern period, $k_{0}x\sin\theta$ should experience a $2\pi$ change. Let it be 0 at the beginning of period, we have

$J_{1}\text{\textasciitilde}\left[\begin{matrix} 1+\cos\theta\\ 2i \end{matrix}\right]$ (S3)

This is a polarization very close to LCP for a small value of *θ*. At the center of period, we have

$J_{2}\text{\textasciitilde}\left[\begin{matrix} 1-\cos\theta\\ 0 \end{matrix}\right]$ (S3)

This is a linear polarization, with very small light intensity for a small value of *θ*. It can be easily observed that for *θ* approaching zero, the whole polarization pattern converges to LCP and the light intensity decreases to zero in period center. But for a small *θ*, the polarization is at first close to LCP state (north pole on Poincaré sphere), and then goes to linear polarization (equator on Poincaré sphere).

**S2 Fabrication of 2-inch template and sample**


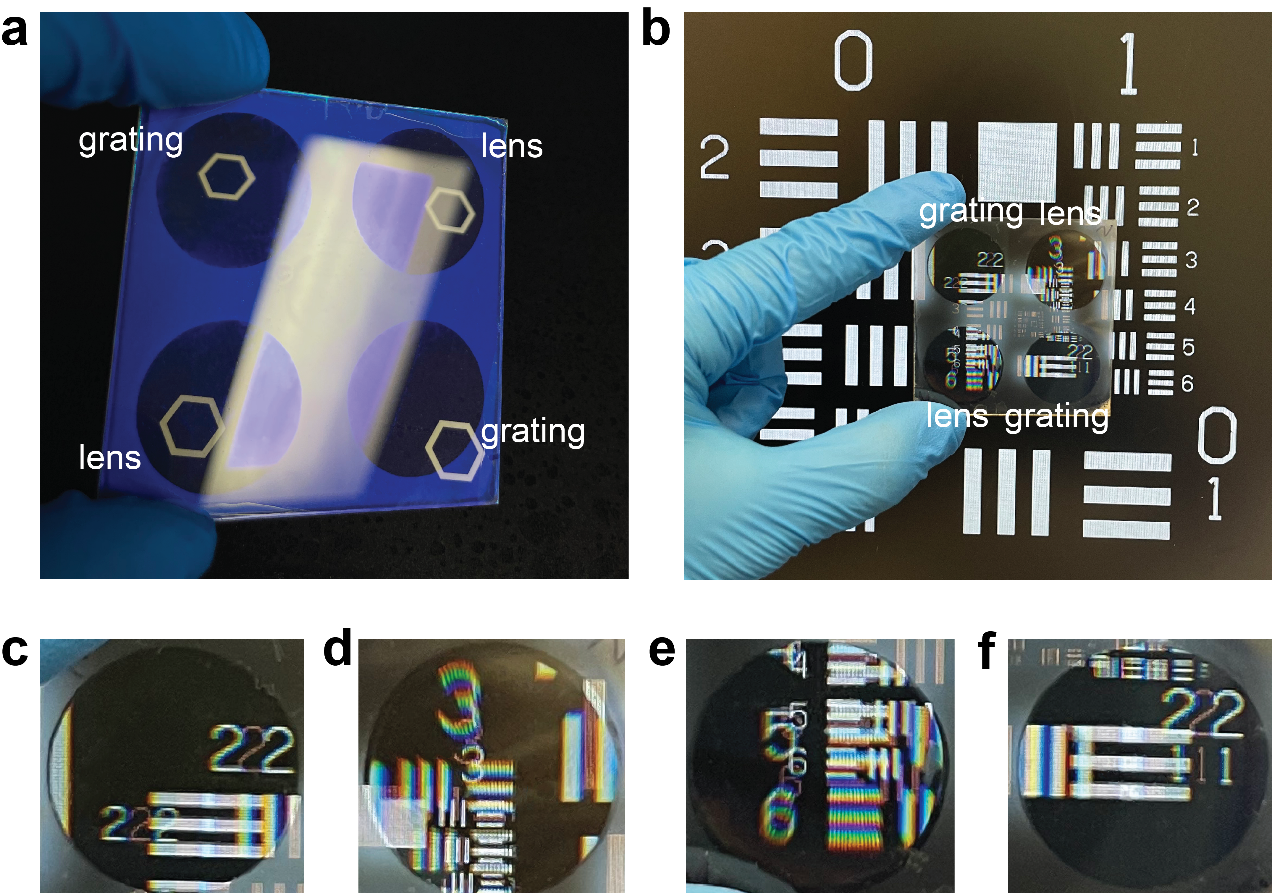


Fig. S2. Fabrication of a 2-inch sample. **a** Photo of the 2-inch template with an array of two lens and grating patterns. The background is four hexagons printed on paper. **b** Photo of the diffraction effect of the holo-imprinted transmissive device. Magnified image of the sample in **c** upper left, **d** upper right, **e** lower left and **f** lower right.

To demonstrate the feasibility of our proposed approach for large-scale fabrication, we fabricate a 2-inch template with a 2-by-2 array of gratings and lenses, as shown in Fig. S2a. The holo-imprinted transmissive sample is shown in Fig. S2b. The background image is a USAF 1951 target shown on a computer monitor. Magnified images on each sample are shown in Figs. S2c to S2f. The light leakage is more obvious than the small-size samples in the article. This is due to the film non-uniformity from the spin-coating process, which is more obvious for margin areas in large samples. However, the film uniformity can be improved with many methods suitable for mass production, including spray coating, dip coating, ink-jet printing and so forth.

**S3 Diffraction properties of transmissive samples**


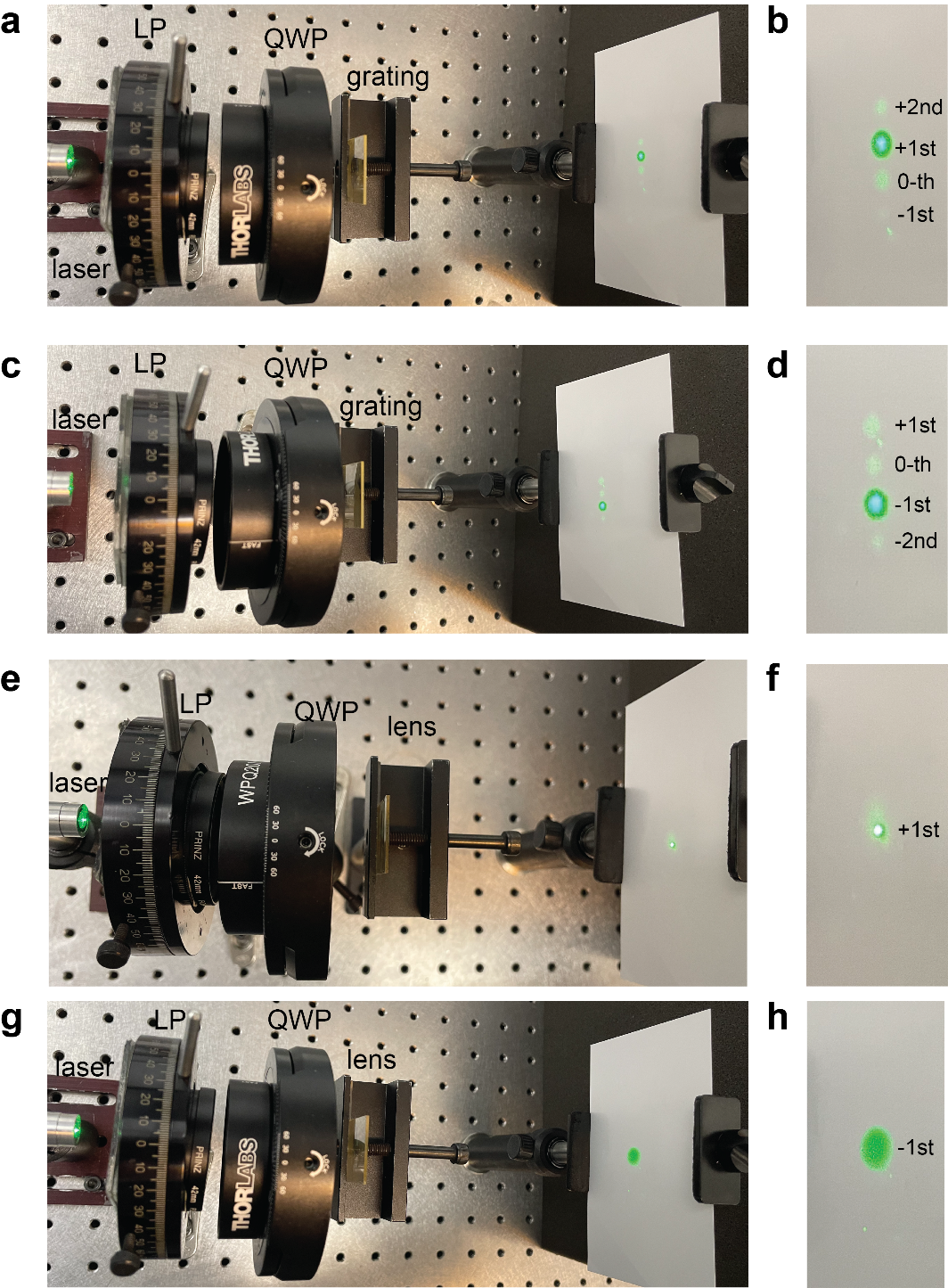


Fig. S3. Diffraction properties of transmissive sample. The laser passes linear polarizer (LP) and quarter-wave plate (QWP) to be converted into CP light. Experiment setup for grating with **a** LCP and **c** RCP light input. Magnified image of diffraction orders for grating for **b** LCP and **d** RCP input. Experiment setup for lens with **e** LCP and **g** RCP light input. Magnified image of diffraction orders for lens for **f** LCP and **h** RCP input.

To illustrate the diffraction properties of transmissive lens and grating samples, we use the experimental setup shown in Fig. S3. The light from a green laser diode (532 nm) firstly passes a linear polarizer, then a QWP and finally the sample. The QWP is rotated so that the light is converted to LCP and RCP lights. The LCP light (Figs. S3a and S3b) is mainly diffracted into the +1^st^ order, and RCP (Figs. S3c and S3d) into -1^st^ order, with negligible leakage into other diffraction orders. The direct measurement of light intensities of all orders gives a diffraction efficiency of 95.6%. This measurement, however, is not as precise as the measurement using the spectrometer, because the QWP is designed for blue light (457 nm). The state of CP light is therefore imperfect and would cause a more serious light leakage. For the lens sample, most of the light is diffracted into the +1^st^ order for LCP light (Figs. S3e and S3f), which forms a tiny focal spot. For RCP light (Figs. S3g and S3h), the lens operates in a diverging mode, with most of the light diffracted into the -1^st^ order, forming a large spot. The special property of the lens leads to the hard observation of other diffraction orders, because other orders form larger spot sizes with very small light intensities. The efficiency is measured to be ~ 100%. Still, this measurement only serves as a rough reference. The spectrometer measurement in the articles has a higher precision.

**Table S1 Parameters of gratings in the simulation**

|  | | Precursor composition | | | | Precursor: toluene  (weight ratios) |
| --- | --- | --- | --- | --- | --- | --- |
|  |  | RM257 | S5011 | Zonyl 8857A | Irgacure  651 |  |
| Large-angle  (Reflective) | Template | 92.35% | 2.55% | 0.1% | 5% | 1:2.5 |
|  | B Sample | 92.28% | 2.62% | 0.1% | 5% | 1:3 |
|  | G Sample | 92.77% | 2.13% | 0.1% | 5% |  |
|  | R Sample | 93.02% | 1.88% | 0.1% | 5% |  |
| Small-angle  (Transmissive) | Template | 92.28% | 2.62% | 0.1% | 5% | 1:2.5 |
|  | Sample | 94.90% | 0 | 0.1% | 5% | 1:3 |
